# Mining Xanthine Oxidase Inhibitors from an Edible Seaweed *Pterocladiella capillacea* by Using In Vitro Bioassays, Affinity Ultrafiltration LC-MS/MS, Metabolomics Tools, and In Silico Prediction

**DOI:** 10.3390/md21100502

**Published:** 2023-09-22

**Authors:** Yawen Wang, Longjian Zhou, Minqi Chen, Yayue Liu, Yu Yang, Tiantian Lu, Fangfang Ban, Xueqiong Hu, Zhongji Qian, Pengzhi Hong, Yi Zhang

**Affiliations:** 1 Guangdong Provincial Key Laboratory of Aquatic Product Processing and Safety, Guangdong Provincial Engineering Laboratory for Marine Biological Products, Guangdong Provincial Center for Modern Agricultural Scientific Innovation, Shenzhen Institute of Guangdong Ocean University, Zhanjiang Municipal Key Laboratory of Marine Drugs and Nutrition for Brain Health, Research Institute for Marine Drugs and Nutrition, College of Food Science and Technology, Guangdong Ocean University, Zhanjiang 524088, China; yavin_wang@163.com (Y.W.); zhoulongjian@gdou.edu.cn (L.Z.); katelyn@outlook.com (M.C.); yayue_liu@163.com (Y.L.); yangyu515900@163.com (Y.Y.); lutiantiana@163.com (T.L.); banfangfang@126.com (F.B.); hxw247@163.com (X.H.); zjqian78@163.com (Z.Q.); hongpengzhi@126.com (P.H.); 2 Southern Marine Science and Engineering Guangdong Laboratory (Zhanjiang), Zhanjiang 524088, China; 3 Collaborative Innovation Center of Seafood Deep Processing, Dalian Polytechnic University, Dalian 116034, China

**Keywords:** *Pterocladiella capillacea*, xanthine oxidase inhibitors, anti-inflammatory, affinity ultrafiltration LC-MS/MS, molecular networking

## Abstract

The prevalence of gout and the adverse effects of current synthetic anti-gout drugs call for new natural and effective xanthine oxidase (XOD) inhibitors to target this disease. Based on our previous finding that an edible seaweed *Pterocladiella capillacea* extract inhibits XOD, XOD-inhibitory and anti-inflammatory activities were used to evaluate the anti-gout potential of different *P. capillacea* extract fractions. Through affinity ultrafiltration coupled with liquid chromatography tandem mass spectrometry (LC-MS/MS), feature-based molecular networking (FBMN), and database mining of multiple natural products, the extract’s bioactive components were traced and annotated. Through molecular docking and ADMET analysis, the possibility and drug-likeness of the annotated XOD inhibitors were predicted. The results showed that fractions F4, F6, F4-2, and F4-3 exhibited strong XOD inhibition activity, among which F4-3 reached an inhibition ratio of 77.96% ± 4.91% to XOD at a concentration of 0.14 mg/mL. In addition, the *P. capillacea* extract and fractions also displayed anti-inflammatory activity. Affinity ultrafiltration LC-MS/MS analysis and molecular networking showed that out of the 20 annotated compounds, 8 compounds have been previously directly or indirectly reported from seaweeds, and 4 compounds have been reported to exhibit anti-gout activity. Molecular docking and ADMET showed that six seaweed-derived compounds can dock with the XOD activity pocket and follow the Lipinski drug-like rule. These results support the value of further investigating *P. capillacea* as part of the development of anti-gout drugs or related functional foods.

## 1. Introduction

Hyperuricemia is a chronic metabolic disease characterized by elevated serum uric acid levels due to long-term purine metabolic disorder or decreased uric acid excretion in the body [1,2]. The long-term high concentration of serum uric acid leads to the crystallization of uric acid in joints and soft tissues in the body, causing damage to connective tissues and triggering gout [3,4]. In acute gouty arthritis, the interaction between urate crystals and phagocytes (such as macrophages and infiltrating white blood cells) induces the secretion of various inflammatory mediators, including cytokines, chemokines, and interleukin, triggering typical inflammatory reactions [5,6]. Therefore, the usual means of treating gout aim to inhibit the production of uric acid and promote the excretion of uric acid. Xanthine oxidase (XOD, EC 1.17.3.2) is the key enzyme involved in the metabolism of human purines into uric acid. It exists in the liver, intestine, serum, and lactating breast, catalyzing the gradual hydroxylation of hypoxanthine into xanthine, and then into uric acid [7]. Its activity directly determines the rate of uric acid formation to a certain extent. Therefore, this target enzyme has received much attention in the treatment of or intervention in cases of hyperuricemia and gout.

Studies have shown that inhibiting the catalytic activity of xanthine oxidase can effectively reduce the production of uric acid, making this an important means to relieve and treat hyperuricemia and gout in the clinic [8]. Allopurinol [9], febuxostat [10], and topiroxostat [11] are the most commonly used xanthine oxidase inhibitors in clinical practice for hyperuricemia and gout. However, these synthetic inhibitors are associated with strong side effects in clinical use, including varying degrees of liver damage, neurological adverse effects, and others. Allopurinol may even cause ‘allopurinol hypersensitivity syndrome’ [12,13]. Therefore, it is of great significance to find new XOD inhibitors from natural sources, with strong activity and low toxicity, for the development of new anti-gout functional foods or drugs. Currently, natural xanthine oxidase inhibitors such as apigenin [14,15], quercetin [15,16], galangin [17,18], and myricetine [19,20] have been reported, most of which are found in terrestrial plants. However, their xanthine oxidase inhibition activity is lower than that of positive drugs, which may hinder their further research and development.

The ocean is a vast treasury of medicinal and edible biological resources, among which seaweeds have the advantage of a huge biomass and cultivability for sustainable development. Till now, there have been several reports on anti-gout active substances including XOD inhibitors derived from seaweeds. For instance, vine alkaloid isolated from *Caulerpa prolifera* has an irreversible XOD-inhibitory effect with an IC_50_ value of 26.92 μM [21]. The fucoidan from *Laminaria japonica* was found to completely reverse the negative changes induced by adenine in mice, restoring the activities of adenosine deaminase (ADA) and XOD in the liver to normal levels [22], which can effectively reduce the serum uric acid content and blood uric acid content of hyperuricemia mice and rats [23]. The *Enteromorpha prolifera* polysaccharide significantly reduced serum uric acid (UA), serum blood urea nitrogen, and serum and hepatic XOD, and also improved histological parameters in hyperuricemic mice [24]. These findings suggest that seaweeds are a meaningful avenue for XOD inhibitor exploration.

For highly efficient discovery of enzyme inhibitors, affinity ultrafiltration mass spectrometry (UF-LC/MS) is a powerful tool that has been increasingly used in screening of bioactive compounds from natural products. In the process of biological affinity ultrafiltration, the ligand–enzyme complexes are retained by the ultrafiltration membrane from the mixture, and then, the ligands released from the complex in the next step of treatment are further identified and quantified using high-performance liquid chromatography–mass spectrometry (HPLC-MS) analysis to achieve rapid identification of bioactive molecules from complex mixtures. Compared with the traditional separation-dependent procedure of active ingredients discovery from natural medicinal plants, UF-LC/MS greatly reduces the cost in terms of time, samples, and expensive reagents [25]. Furthermore, several metabolomics tools have been developed to automate secondary metabolite identification such as MSDAIL [26], MSFINDER [27], Global Natural Products Social Molecular Networking (GNPS) [28], and its updated version, feature-based molecular networking (FBMN) [29,30]. Previously, we presented a combined strategy named Bio-LCMS-GNPS to connect UF-LC/MS and GNPS, which provided a new approach to enzyme inhibitor discovery from terrestrial and marine bioresources [31].

*Pterocladiella capillacea* (S.G. Gmelin) belongs to the family of Pterocladiaceae, the order of Gelidiales, and the Phylum of Rhodophyta. It is mainly found in tropical and sub-tropical waters and partially inhabits temperate zones. As an edible seaweed plant, *P. capillacea* has been traditionally used as a source of jelly production in eastern Asian countries [32]. Currently, reports have appeared on the antibacterial [33], antioxidant [34], and bacterial cell agglutination potential [35] of *P. capillacea*. In a preliminary study based on local seaweeds in Zhanjiang, China, the crude extracts of *P. capillacea* exhibited strong xanthine oxidase-inhibitory activity. To explore the value of this edible seaweed as anti-gout drugs or functional foods, we further evaluated the XOD-inhibitory and anti-inflammatory activities of its fractions, traced the possible XOD inhibitors via UF-LC-MS/MS, annotated them using metabolomics tools including MSDIAL, MSFINDER, and FBMN, and predicted the action mechanism and drug-likeness of the annotated XOD inhibitors via molecular docking and ADMET, as described in this paper.

## 2. Results

### 2.1. Evaluation of XOD Inhibition Activity

**Figure 1:**
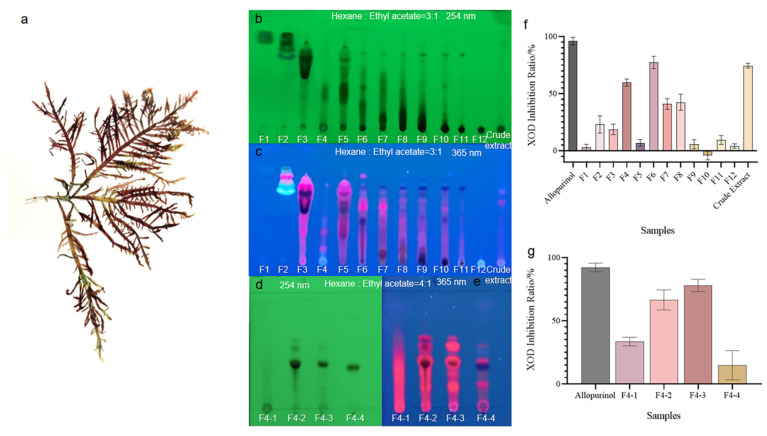
The TLC fingerprints and XOD-inhibitory activity of the crude extract and fractions of *Pterocladiella capillacea*. (**a**) The image of the seaweed material *P. capillacea.* (**b**–**e**) The TLC images of fractions F1–F12, F4-1–F4-4, and crude extract detected under 254 nm and 365 nm UV light, respectively (sample numbers are marked below the start line). (**f**,**g**) The XOD inhibition rates of fractions F1–F12, F4-1–F4-4, crude extract, and allopurinol.

The *P. capillacea* samples were collected along the seashore of Naozhou Island, Zhanjiang, China (see voucher specimens in Figure 1a). Its crude extract was prepared by anhydrous ethanol extraction using an air-dried seaweed sample. Then, the extract was fractioned by a silica gel column to produce twelve primary fractions F1–F12, as shown in the thin-layer chromatography (TLC) images (Figure 1b,c). The XOD inhibition activity screening indicated that fractions F4 and F6 ranked as the top two for activity (Figure 1f). Considering the relative simplicity of the fingerprint and the relatively higher amount of F4 compared with F6, this fraction was preferentially chosen for further study. The separation of F4 on Sephadex LH-20 gel column yielded four secondary fractions, F4-1 to F4-4 (Figure 1d,e). Among them, F4-2 and F4-3 displayed the top two highest XOD inhibition ratios (IRs: 66.47% ± 7.96% and 77.96% ± 4.91%, respectively) at the final concentration of 0.14 mg/mL (Figure 1g). At the same dose, the positive control, allopurinol showed an IR of 92.21% ± 3.38%.

### 2.2. Evaluation of Anti-Inflammatory Activity

Since xanthine oxidase catalyzes the production of urea from xanthine and hypoxanthine, generating a large amount of hydrogen peroxide and superoxide anion, it is possible that substances with xanthine oxidase-inhibitory activity may also exhibit anti-inflammatory activity, and many studies have confirmed this hypothesis [36,37,38]. Considering that inflammation can severely influence the progress of gout, the XOD-inhibitory samples F4 and F6, and the crude extracts found in the preliminary screening, were also evaluated for their anti-inflammatory activity.

**Figure 2:**
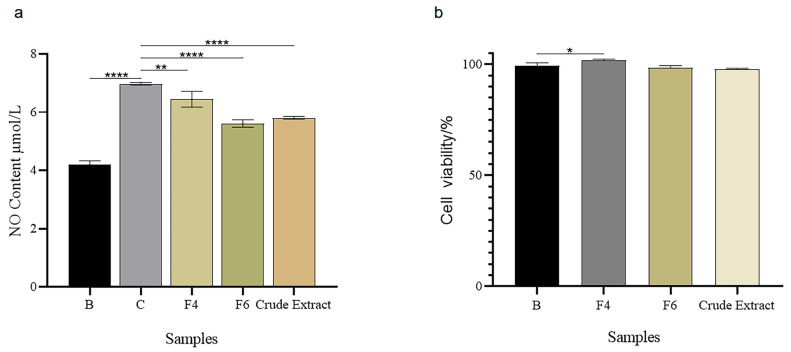
Anti-inflammatory activity and cell viability of XOD-inhibitory samples including F4, F6, and the crude extract. (**a**) The control group (C) was RAW264.7 cells stimulated with lipopolysaccharide (LPS). The sample groups (F4, F6, and crude extract) were RAW264.7 cells stimulated with LPS (dose: 1 μg/mL) and treated with the samples (dose: 20 μg/mL). (**b**) The blank group (B) was cells with viability of RAW 264.7 without treatment. The sample groups (F4, F6, and crude extract) were cells with viability of RAW 264.7 and treated with the samples (dose: 20 μg/mL). Data were expressed as mean ± SD (*n* = 3). **** *p* < 0.001, vs. control; ** *p* < 0.01, vs. control; * *p* < 0.05, vs. blank.

As shown in Figure 2a, the NO production in the RAW264.7 cells of the control group (C) significantly increased compared with the blank group (B) (*p* < 0.001) under the stimulus of lipopolysaccharide (LPS), while samples F4 and F6, and the crude extract can significantly decrease the NO production at a dose of 20 μg/mL. Meanwhile, F4, F6, and the crude extract did not show toxicity to the cells at this dose (Figure 2b). This result was consistent with that of XOD inhibition activity, indicating that these samples also possess anti-inflammatory activity.

### 2.3. UF-LC-MS Screening of XOD Ligands in P. capillacea Extract

To trace the active molecules targeting XOD in active secondary fractions F4-2 and F4-3, affinity ultrafiltration-LC-MS/MS analyses were performed.

After the affinity ultrafiltration treatment, a preliminary HPLC analysis was conducted to check the capturing effect of the enzyme on the possible ligands contained in the samples. This was based on the hypothesis that compounds specifically bound to XOD should exhibit higher peaks in the process groups (P) incubated with XOD than in the corresponding blank groups (B) which were incubated with the inactivated enzyme. Indeed, larger peaks of the captured ligands were observed in the chromatograms of both F4-2 and F4-3 after affinity ultrafiltration treatment.

**Figure 3:**
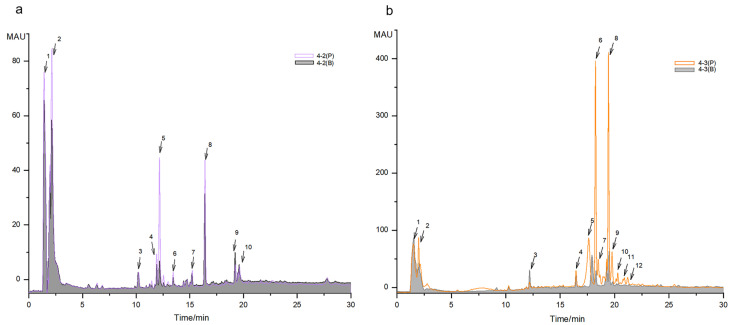
The comparison of HPLC traces of the ultrafiltration membrane-retained samples after incubation with XOD or inactivated XOD ultrafiltration (the chromatograms were monitored under 290 nm). (**a**) The comparison of affinity ultrafiltration process group (4-2(P)) and inactivated enzyme blank group (4-2(B)) for sample F4-2. (**b**) The comparison of affinity ultrafiltration process group (4-3(P)) and inactivated enzyme blank group (4-3(B)) for sample F4-3. The numbers associated with arrows marked the main peaks.

Among them, the peak heights of peak 1, peak 2, peak 5, and peak 8 of F4-2 in the process group 4-2(P) after affinity ultrafiltration treatment were remarkably higher than those of the inactivated enzyme blank group 4-2(B) (Figure 3a). More dramatically, in sample F4-3, more peaks, including peaks 5, 6, 7, 8, 9, 10, 11, and 12, were observed in the process group 4-3(P), with much stronger intensity than in the blank group 4-3(B) (Figure 3b). The peaks were assumed to be the substances in *P. capillacea* that specifically interact with xanthine oxidase.

### 2.4. Comparison of Metabolite Profiles by LC-MS/MS and Multiple Database Mining

**Figure 4:**
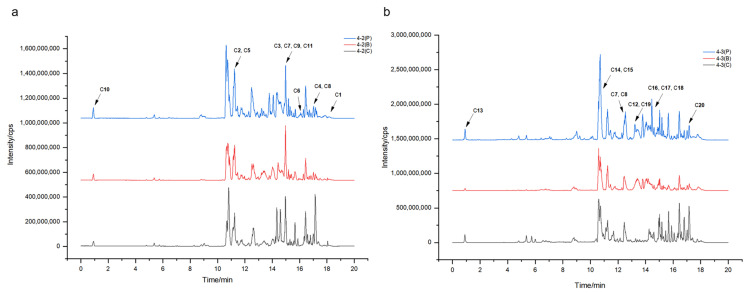
The LC-MS traces (base peak chromatographies, BPC) for the fraction samples 4-2 and 4-3 under positive ion mode of the ultrafiltration membrane-retained samples after incubation with XOD or inactivated XOD ultrafiltration (the chromatograms were monitored under 290 nm). (**a**) The comparison of affinity ultrafiltration process group (4-2(P)), inactivated enzyme blank group (4-2(B)), and crude sample group (4-2(C)) for sample F4-2. (**b**) The comparison of affinity ultrafiltration process group (4-3(P)), inactivated enzyme blank group (4-3(B)), and crude sample group (4-3(C)) for sample F4-3.

To further characterize the possible XOD ligands in F4-2 and F4-3, the above samples for groups 4-2(B), 4-2(P), 4-3(B), and 4-3(P) were analyzed using Orbitrap LC-MS/MS. The data were aligned via MS-DIAL to accurately localize the XOD ligands based on peak area comparison between different treatment groups, obtain their chromatographic and mass spectroscopic information (retention times, molecular weights, molecular formulae, and MS/MS spectra), and annotate them via FBMN, MSDIAL, and MSFINDER. In addition, the sources of compounds were manually screened by searching open and accessible natural product databases, including PubChem, Natural Product Dictionary (DNP), NPASS, LOTUS, COCONUT, and The Natural Products Atlas. As shown in Figure 4 and Table 1 and Table 2, a total of 20 compounds from fractions F4-2 and F4-3 were identified as XOD ligands based on their much higher peak areas in the affinity ultrafiltration process groups (“P” groups) than in the corresponding enzyme inactivated blank groups (“B” groups). Compounds **1**–**11** were from F4-2, compounds **12**–**20** were from F4-3, and compounds **7**–**8** were from both (Figure 4).

**Figure 5:**
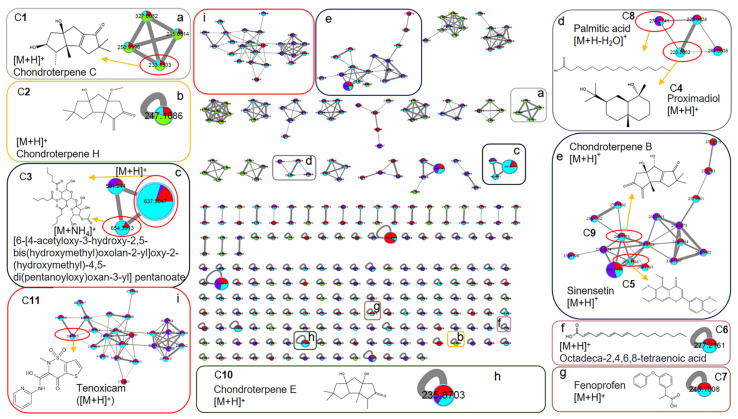
The FNMB molecular network for the fraction sample 4-2 based on positive ion MS/MS spectral similarity. Sub-figures (**a**–**i**) show the details of the amplified clusters including the annotated compounds **1**–**11** (C**1**–C**11**), respectively. The nodes display the measured average masses of the molecular ions with identical MS/MS spectra. The sizes of the nodes reflect the relative amount of the corresponding compounds. The different colors of sections in the nodes represent different sample groups, i.e., 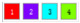: 4-2(C) (Group1), 4-2(B) (Group 2), 4-2(P) (Group 3), and Blank (Group 4).

**Figure 6:**
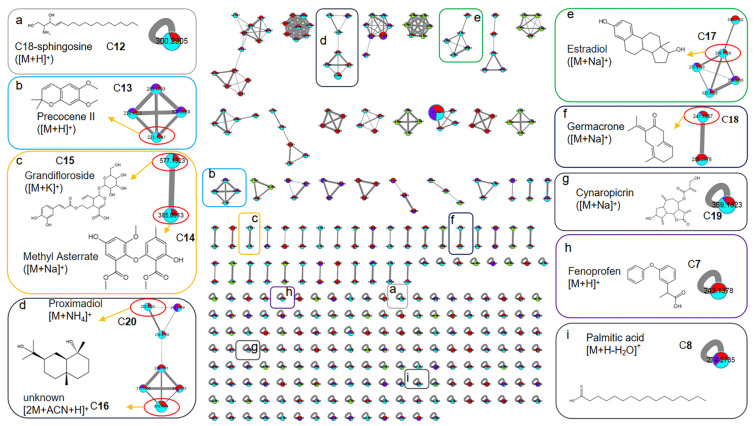
The FBMN molecular network for the fraction sample 4-3 based on positive ion MS/MS spectral similarity. Sub-figures (**a**–**i**) show the details of the amplified clusters including the annotated compounds **7**, **8**, and **12**–**20** (C**7**, C**8**, and C**12**–C**20**), re-spectively. The nodes display the measured average masses of the molecular ions with identical MS/MS spectra. The sizes of the nodes reflect the relative amount of the corresponding compounds. The different colors of sections in the nodes represent different sample groups, i.e., 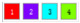: 4-3(C) (Group 1), 4-3(B) (Group 2), 4-3(P) (Group 3), and Blank (Group 4).

For these ligands, the MSDIAL-MSFINDER-FBMN pipeline provided some annotated structures through MS/MS matching. However, manual searching in natural product databases showed that most of these structures were not from taxonomically close sources (Order of Gelidiales or Phylum of *Rhodophyta*). It was deduced that the MS/MS spectral libraries of MSDIAL, MSFINDER, and FBMN had not indexed a sufficient amount of records from this taxon. Thus, the molecular weight (MW) and molecular formula (MF) information of the annotations were used to search the natural product databases as well. And the taxonomical range was restricted to the Order of Gelidiales to obtain the most relevant hits. Finally, 15 compounds were annotated through MS/MS matching combined with MW-MF searching, 4 compounds were annotated through MW-MF searching, and 1 compound remained unknown (compound **16**). The annotation details, including metabolite information, results, methods, biological sources, and reports on anti-gout or anti-inflammatory related activities, are summarized in Table 1 and Table 2 and Figure 5 and Figure 6. The MS/MS spectra of these compounds are provided in the (Supporting Information Figures S1–S21).

Among the 20 annotated compounds (XOD ligands), 8 are natural products derived from seaweeds, including 4 from *P. capillacea* endophytic fungus, and 10 (compounds **1**, **5**, **7**, **8**, **11**, **12**, **15**, **17**, **18**, and **19**) have been reported for anti-gout-related or anti-inflammatory activities, providing scientific support for the XOD-inhibitory and anti-inflammatory activity of the extract and fractions of *P. capillacea*.

Based on MS/MS spectral similarity, the FBMN molecular networks were visualized to display the metabolites in fractions F4-2 (Figure 5 presents ligands **1**–**11**) and F4-3 (Figure 6 presents ligands **7**, **8**, **12**–**20**).

### 2.5. Molecular Docking and ADMET Analysis

Seven annotated compounds with seaweed-related origins but without reports of anti-gout-related activity were predicted to have an affinity for XOD using molecular docking and were then subjected to ADMET drug-likeness analysis. The Molybdopterin domain is the catalytic center of XOD [57], which is used for docking the treated ligand compound and calculate the minimum binding affinity. The lower the minimum binding affinity, the stronger the affinity between ligand and XOD, and the higher the binding stability. The molecular docking scoring results are shown in Table 3.

**Figure 7:**
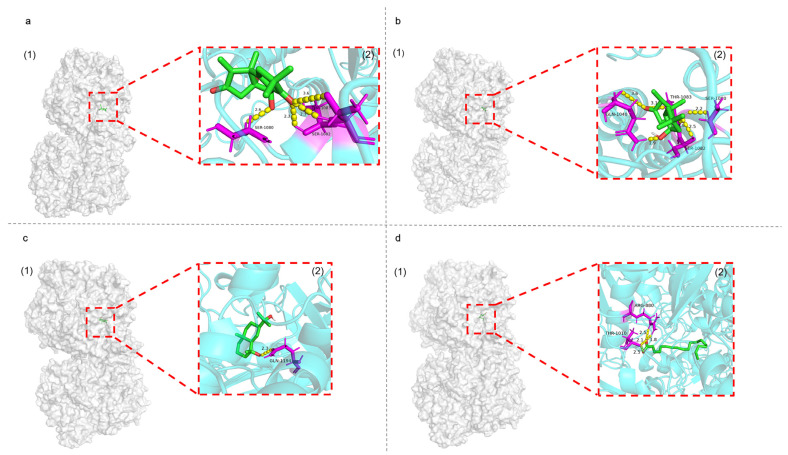
Molecular docking results of Chondroterpene B (**a**), Chondroterpene E (**b**), Proximadiol (**c**), and Octadeca-2,4,6,8-tetraenoic acid (**d**), respectively, with XOD. Docking pose (1) and ligand interaction (2) diagrams of different ligands and XOD are presented for each docking result. Yellow dotted lines indicate hydrogen bonds.

The ranking of the minimum binding affinity shows that the XOD protein binds most stably to Chondroterpene B, followed by Chondroterpene E, Proximadiol, and Octadeca-2,4,6,8-tetraenoic acid. The docking diagram showed that Chondroterpene B enters deeply into the XOD active pocket and forms one hydrogen bond with residues SER-1080 and THR-1083 of the XOD protein and two hydrogen bonds with residue SER-1082, respectively (Figure 7a). Chondroterpene E enters deeply into the XOD active pocket and forms one hydrogen bond with residues SER-1080 and SER-1082 and forms two hydrogen bonds with residues GLN-1040 and THR-1083 each, respectively (Figure 7b). Proximadiol enters deeply into the XOD active pocket and forms a hydrogen bond with residue GLN-1194 of the XOD protein (Figure 7c). Octadeca-2,4,6,8-tetraenoic acid enters into the XOD active pocket and forms two hydrogen bonds with residues ARG-880 and THR-1010 each, respectively (Figure 7d). In addition, the docking results showed that all of the seven compounds except sphingosine enter the XOD activity pocket.

The ADMET properties of a drug are the absorption, distribution, metabolism, excretion, and toxicity of the drug in the human body, and these are key factors used to evaluate whether a compound can become a drug or not. To validate the drug-likeness of the compounds annotated in the active fractions of *P. capillacea*, they were subjected to online ADMET prediction, and the results are shown in Table 4.

Likewise, the seven annotated compounds, except the C_18_-sphingosine, showed suitable water solubility (−4 < LogS < 0.5), excellent absorption in the human small intestine (HIA < 0.3), and low effect on drug metabolism (CYP inhibitor). Octadeca-2,4,6,8-tetraenoic acid and Sphingosine have poor oral bioavailability (PPB > 90%) and may also cross the blood–brain barrier, while the other five compounds have low blood–brain barrier penetration (BBB > 0.7) and therefore do not cause side effects on the central nervous system. Moreover, Chondroterpene C, Chondroterpene H, Proximadiol, and Chondroterpene E may have improved safety characteristics, since they may not cause hepatotoxicity, liver damage, or skin sensitization (H-HT, DILI, Skin Sensitization < 0.3). Generally, all the seven compounds meet the five principles of Lipinski’s rule for oral drugs [58].

## 3. Discussion

In the present study, two extract fractions (F4 and F6) from edible seaweed *P. capillacea* were discovered to have strong XOD-inhibitory and anti-inflammatory activities. From F4, with relatively simple components, two active subfractions, F4-2 and F4-3, were obtained and exhibited remarkable XOD-inhibitory activity. By using UF-LC-MS/MS, metabolomics tools, multiple natural product database mining, molecular docking, and ADMET analysis, 20 plausible XOD ligands in the active subfractions were localized and annotated, and 7 annotated seaweed-derived compounds were further predicted to have binding affinity with XOD and drug-likeness. Among them, Chondroterpene C, Chondroterpene E, and Proximadiol displayed strong XOD affinity and good drug-likeness properties.

Hyperuricemia and gout have become increasingly prevalent globally, while current clinical drugs like allopurinol have severe side effects including triggering ‘allopurinol hypersensitivity syndrome’ [13]. Therefore, there is a need for new effective drugs and functional foods to cope with this issue. *P. capillacea,* a globally spread seaweed that is traditionally eaten in eastern Asia, has not been reported for its anti-gout activity. In this study, potent fractions and subfractions from *P. capillacea* have been found to inhibit XOD activity and inflammation. The potency of *P. capillacea* fractions and subfractions is generally comparable to the control allopurinol at the same dose. Furthermore, the fractions did not show cytotoxicity to macrophage cells. Since XOD is the key target in hyperuricemia and gout, and inflammation also plays an important role in the development of gout [36], *P. capillacea*, as an edible seaweed with huge biomass, may have great potential as a new source for the development of anti-hyperuricemia and anti-gout drugs or functional foods.

Preliminary UF-LC-MS/MS tracing, annotating, and predictive investigations located the active peaks and suggested 20 compounds as plausible XOD inhibitors in *P. capillacea*, including 8 compounds with seaweed and *P. capillacea*-related origins, and 9 compounds with XOD-inhibitory or anti-inflammatory reports. Furthermore, molecular docking of seven compounds with seaweed and seaweed-related origins and no reported anti-gout effects showed that six of them enter the XOD activity pocket and dock with XOD by forming hydrogen-bonding forces with amino acid residues. Three compounds, Chondroterpene C, Chondroterpene E, and Proximadiol, displayed strong XOD affinity and good drug-likeness properties. To some extent, the above results have provided reasonable explanations for the XOD-inhibitory and anti-inflammatory activity of *P. capillacea* extracts and useful clues for future studies on natural product research for anti-gout treatments using this seaweed.

In addition, it is intriguing that compounds **1** (Chondroterpene C), **2** (Chondroterpene H), **10** (Chondroterpene B), and **11** (Chondroterpene E) are hirsutane-type sesquiterpenes, which are reported to be isolated from *P. capillacea* endophytic fungus *Chondrostereum* sp. NTOU4196 [39]. Until now, all reported hirsutane-type sesquiterpenes have been identified from terrestrial and marine Basidiomycotina, especially from Agaricomycetes including genera/species like *Stereum hirsutum* [59,60], *Chondrostereum* sp. [61,62], *Cerrena* sp. [63], etc. Seaweed *P. capillacea* has been reported to be a rich source of endophytic fungi, including four genera belonging to Agaricomycetes, i.e., *Chondrostereum* sp., *Cerrena* sp., *Bjerkandera* sp., and *Grammothele* sp. [64], two of which are reported to be hisrutane-producing genera. Considering the fact that many natural plant products like Taxol, Camptothecin, and Quinine are produced by the endophytic fungi [65], it is reasonable to suggest that the plausible Chondroterpenes detected in *P. capillacea* are possibly produced by its endophytes.

UF-LC-MS/MS has been increasingly used in enzyme inhibitor discovery, where the LC-MS profile of an affinity-treated enzyme group is usually compared with that of the blank group without enzyme incubation to recognize the ligands specifically binding with the enzyme [66,67,68]. In the blank group, the molecules absorbed by the ultrafiltration membrane are taken as non-specific binders. However, it is not easy to discriminate the molecules absorbed by the surface of the proteins using this method. In this study, the authors used heat-inactivated enzymes for the incubation treatment as a blank group instead of only ultrafiltration membrane, by which the small molecular ligands that specifically interact with the target enzyme may be more accurately recognized from complex natural product mixtures. Although LC-MS/MS can provide rich information about the samples, in this study, a preliminary HPLC-DAD analysis was performed for the ultrafiltration experimental samples before formal LC-MS/MS analysis. This measurement can not only verify the effect of ultrafiltration experiments, but also provide more spectrometric information on the active peaks, in addition to mass spectra for further natural product purification, since HPLC-DAD is a more frequently used approach in regular analytic tasks.

Bioactivity-coupled molecular networking analysis can rationalize natural product isolation and help in deduplication before time-consuming isolation tasks. Compared with our previously reported “Bio-LCMS-GNPS” strategy used in acetylcholinesterase inhibitors and antioxidants mining [31], improvements have been made in two aspects of the present study. Firstly, FBMN has been used to construct molecular networks instead of classical GNPS, and more metabolomics tools and multiple natural product databases are utilized to annotate ligands together. Since high-resolution mass spectrometry-based FBMN provides molecular formulae, more accurate peak area integration, and improved retention times for the features, and recognize different adduct ions of the same substance [69], it has an advantage in comparing peak areas of ligands between different groups and providing molecular formulae for library searching over classical GNPS. FBMN combined with MSDIAL, MSFINDER, and natural products databases, also provides more comprehensive annotation than GNPS individually. Secondly, following the annotation, in silico molecular docking and ADMET were performed to evaluate the possibility of annotated compounds as enzyme ligands and their drug-likeness, which is more reliable than mere metabolomics annotation. Therefore, this updated bioactivity-coupled mass spectrometric metabolomics pipeline is named “Bio-LCMS-Metabolomic-in silico Prediction”.

The application of this pipeline to the discovery of XOD inhibitors in *P. capillacea* has not only provided plausible explanations for the bioactivity of samples, but has also located the XOD ligands in the samples. The feature information, including retention time, molecular weights, MS/MS spectra, and UV spectra, will guide future compound isolation and elucidation. As with all the MS metabolomics-based studies, the isolation, structural elucidation, and bioactivity study of isolated pure compounds is the final verification and “golden criterion” for the annotated results. This study and ongoing similar processes on fraction F6 will guide our in-depth chemistry and biology studies on the anti-gout constituents of *P. capillacea* for drugs and functional foods purposes.

In conclusion, the present study has revealed the great potential of the edible seaweed *P. capillacea* for developing anti-hyperuricemia and anti-gout drugs and functional foods. Furthermore, it has established a new bioactivity-coupled metabolomics investigating pipeline “Bio-LCMS-Metabolomic-in silico Prediction” for bioactive natural product discovery.

## 4. Materials and Methods

### 4.1. Materials and Chemicals

*P. capillacea* was harvested from Naozhou Island, Zhanjiang, China, in July of 2020. The seaweed was identified by an algologist, Prof. Enyi Xie, at Guangdong Ocean University, and a voucher specimen was deposited in our laboratory with number ZJNZ2020-2. Xanthine oxidase (EC 1.17.3.2, 5 U, Sigma, Saint Louis, MO, USA), allopurinol (98% purity, 25 g, Aladdin, Shanghai, China), xanthine (analytical purity, 1 g, Solabao, Beijing, China), Tris-HCL (pH 8.7, 0.5 mmol/L, Solabao, Beijing, China), 96-well UV plate (Model 3635, Corning, Kennebunk, ME, USA), ultrafiltration centrifuge tube (0.5 mL, 100 Kd, Millipore, Billerica, MA, USA), nitric oxide detection kit (Beyotime, Shanghai, China), Cell Counting Kit-8 (CCK-8, Zeta Life, Menlo Park, NJ, USA) and RAW264.7 cell line (Cell Bank of Chinese Academy of Sciences, Shanghai, China) were used in the bioassays. All organic mobile-phase solvents used for LC-MS and the thin-layer chromatographic plate (silica gel 60 F254) were from Merck (Darmstadt, Germany). The materials for column chromatography (CC) included silica gel (100–200 mesh, Qingdao Marine Chemistry Co., Ltd., Qingdao, China) and Sephadex LH-20 (GE Healthcare Biosciences AB, Stockholm, Sweden). All other reagents were of analytical purity.

An Agilent 1200 high-performance liquid chromatography and a Thermo Orbitrap Fusion LUMOS Tribrid liquid chromatography–mass spectrometer (Orbitrap LC-MS/MS, Thermo Fisher Scientific, Waltham, MA, USA) were used to analyze the samples. A 96-well microplate reader (Bio-Tek Epoch 2, Bio Tek Instruments, Winooski, VT, USA) was used for spectrophotometric measurements. An Allegra X-30R high-speed centrifugator (Beckman Coulter, Brea, CA, USA) was used for ultrafiltration experiment.

### 4.2. Preparation of Crude Extracts of P. capillacea

The air-dried *P. capillacea* (3 kg) was crushed and soaked in anhydrous ethanol for 24 h with a ratio (g:mL) of 1:10, extracted three times, filtered, and concentrated to dryness below 40 °C with a rotary evaporator to obtain the crude extract, and then stored at −20 °C.

### 4.3. Separation of the Crude Extract

First, 13 g of crude extract was separated with a silica gel column (200–300 mesh) which was eluted stepwise using n-hexane/ethyl acetate (1:0 to 0:1) and ethyl acetate/methanol (1:0 to 0:1); the eluates were detected via HPTLC. Twelve primary fractions (F1 to F12) were obtained. The fraction F4 showing strong XOD-inhibitory activity in bioassay and was further separated using a Sephadex LH-20 gel filtration column, eluted with methanol/dichloromethane = 2:1 solution to yield four secondary fractions (F4-1 to F4-4) for further study.

### 4.4. In Vitro Determination of XOD-Inhibitory Activity

The in vitro XOD-inhibitory activities were evaluated with UV transparent 96-well microwell plates using a modified method based on Andriana’s report [70], in which the enzyme activity was reflected by the UV absorbance of uric acid derived from xanthine.

The reaction mixture contained 17.5 μL XOD solution (20 U/L, in phosphate-buffered solution (PBS) containing 0.014 mol/L KH_2_PO_4_, 0.061 mol/L K_2_HPO_4_·3H_2_O, and 0.111 mol/L EDTA-Na_2_ with pH 7.4), 119.5 μL PBS, 3 μL sample solution (10 mg/mL in DMSO), and 70 μL xanthine (0.4 mmol/L in PBS). The microwell plates were incubated at 37℃ for 10 min before absorbance was measured (*OD*_1_) at 295 nm. For control, the sample was replaced by DMSO (*OD*_2_). For sample background subtraction, XOD was replaced by PBS (*OD*_3_). For the blank sample, XOD and sample were replaced by PBS (*OD*_4_). Allopurinol (10 mg/mL in 0.1 mol/L NaOH) was taken as positive control. All samples were tested in triplicate. The inhibition ratios were calculated using the following formula:

(1) $$I(\%)=(1-\frac{{OD}_{1}-{OD}_{3}}{{OD}_{2}-{OD}_{4}})\times 100\%,$$

### 4.5. Cellular Anti-Inflammatory Activity Assay

The content of NO in cells and cell survival rate were determined in accordance with a previously reported method [71]. In accordance with the manufacturer’s protocol, the NO level of RAW264.7 cells treated with or without lipopolysaccharide and *P. capillacea* extracts (crude extract, F4 and F6) was evaluated using the Griess reagent system. The viability of RAW264.7 cells treated with or without *P. capillacea* extracts (crude extract, F4 and F6) was evaluated via CCK-8.

RAW264.7 cells were seeded in a 24-well plate at a density of 5 × 10^4^ cells/well. In all the test groups, the cells were treated with the extracts (crude extract, F4 and F6) of *P. capillacea* with a concentration of 20 μg/mL. Subsequently, they were activated with LPS (1 μg/mL) for 24 h, except for the blank group. Finally, NO production was estimated via spectrophotometry at 540 nm.

Cell viability assay: RAW264.7 cells were inoculated in 96-well plates at a density of 5 × 10^4^ cells/well for 24 h. *P. capillacea* extract (crude extract, F4 and F6) was added to reach a final concentration of 20 μg/mL and incubated for 24 h. After 10 μL of CCK-8 solution was added to each well for 1 h, absorbance values were measured at 450 nm.

### 4.6. UF-LC-MS/MS

#### 4.6.1. Affinity Ultrafiltration Treatment

First, 10 mg of sample (in 100 μL methanol) was applied onto a reverse SPE column, eluted with 1 mL pure water to remove salts, and then with 1 mL methanol to collect the eluent. This eluent solution was then filtrated through 0.22 μm membrane, dried completely, and finally dissolved in 20 μL of DMSO plus 80 μL of water to form the sample solution used for affinity ultrafiltration. For the process group (P), 50 μL of sample solution, 50 μL of 0.5 U/mL of XOD solution, and 100 μL of Tris-HCL buffer were added to a 1.5 mL centrifuge tube and incubated at 37 °C for 30 min. Afterwards, the unbound small molecules were removed by centrifugation at 11,200× *g* for 10 min and repeatedly washed using Tris-HCL buffer and centrifugation 3 times. Then, the ligands were released from the retained ligand–enzyme complexes on the ultrafiltration membrane using 100 μL of methanol-water (4:1) (centrifugation at 11,200× *g* for 10 min, repeated 3 times). The eluate was dried and dissolved in 1 mL of LC-MS pure methanol for HPLC and Orbitrap LC-MS/MS analyses. For the blank group (B), the difference was that the XOD solution was denatured at 100 °C for 30 min before use. For the crude sample group (C), the sample was prepared directly to 5 mg/mL after desalting, without ultrafiltration.

#### 4.6.2. HPLC Preliminary Analysis

The HPLC analyses of samples were performed on an Agilent 1200 HPLC instrument connected with a diode array detector (DAD). The columns were Phenomenex Kinetex C18 100A (Phenomenex, Torrance, CA, USA) reversed-phase columns (100 × 4.60 mm, 5 µm). The injection volume was 20 µL. The gradient mobile phase was: 30% MeCN/H_2_O for 2 min, 30% MeCN/H_2_O to 95% in 12 min, 95% MeCN/H_2_O for 15 min, 95% MeCN/H_2_O to 30% MeCN/H_2_O in 0.2 min, and finally 30% MeCN/H_2_O for 2.8 min with a flow rate of 0.6 mL/min. The MeCN was added with 0.1% HCOOH.

#### 4.6.3. LC-MS/MS Analysis

LC–MS/MS analysis was performed on a Thermo Orbitrap Fusion LUMOS Tribrid liquid chromatography–mass spectrometer, and a Waters Acquity UPLC BEH C18 column (1.7 µm, 2.1 × 100 mm, Waters, Milford, CT, USA) was used for analysis. The injection volume was 10 µL. The gradient mobile phase was: 10% MeCN/H_2_O for 1 min, 10% MeCN/H_2_O to 60% in 9 min, 60% MeCN/H_2_O to 90% in 3 min, 90% MeCN/H_2_O for 3 min, then 90% MeCN/H_2_O to 10% MeCN/H_2_O in 0.2 min, and finally 10% MeCN/H_2_O for 3.8 min with a flow rate of 0.3 mL/min. Both the water and the MeCN contained 0.1% HCOOH. Mass spectra were recorded in positive ESI mode (*m*/*z* 200–2000) and with an automated fully dependent MS/MS scan enabled.

### 4.7. Annotation of the Bioactive Molecules Using Metabolomics Tools

The standard pipeline of feature-based molecular networking (FBMN) with MS-DIAL was performed by referring to a previous report [29] and following the instructions on the webpage of the GNPS platform (https://ccms-ucsd.github.io/GNPSDocumentation/featurebasedmolecularnetworking-with-ms-dial/, accessed on 17 December 2022). The parameters for clustering and compound matching were set as follows: minimum matching fragment of 6; minimum cluster size of 2; cosine threshold of 0.7; and search database range of the entire GNPS library. Data visualization was performed using Cytoscape 3.7.2 software. In addition, the annotation of compounds was also performed via MS-DIAL database matching and MS-FINDER matching with all its indexed natural product databases [72,73].

Furthermore, to retrieve the biological source of the annotated compounds, the following open access natural product databases were searched manually: the PubChem (https://pubchem.ncbi.nlm.nih.gov/, accessed on 15 August 2023), the Dictionary of Natural Products (DNP) (http://dnp.chemnetbase.com/, accessed on 15 August 2023), the NPASS (http://bidd.group/NPASS/, accessed on 15 August 2023), the Natural Product Atlas (https://www.npatlas.org/, accessed on 15 August 2023), and the COCONUT (https://coconut.naturalproducts.net/, accessed on 15 August 2023).

### 4.8. Molecular Docking and ADMET Analysis

The sdf structures of ligands were downloaded from the PubChem database and were batch processed into pdbqt files suitable for molecular docking using openbabel 2.3.2. The crystal structure of XOD protein was obtained from the Protein Database Bank (PDB ID: 3nvw). Water molecules and guanine ligands were removed from the protein using Pymol 2.5.4 software and the protein was exported to pdb format. Then, the protein was hydrogenated and structurally optimized using Autodock vina 1.5.7, and finally exported to pdbqt format. Molecular docking of all ligands to XOD was performed using Autodock vina 1.5.7 and the docking results are visualized using Pymol 2.5.4.

ADMET analysis was performed online on the ADMETlab prediction website (https://admetmesh.scbdd.com/, accessed on 2 April 2023) to predict the adsorption, distribution, metabolism, excretion, and toxicity characteristics of compounds by entering the SMILES codes of the compounds.

### 4.9. Statistical Analysis

All experiments and analyses were performed at least in triplicate, and the obtained results were expressed as “mean ± standard deviation”. Statistical differences of mean values were evaluated using Duncan’s multiple range test using GraphPad Prism 8.0.1 software (San Diego, CA, USA). A probability of less than 5% (*p* < 0.05) was considered significant.

## Figures and Tables

**Table 1:** The annotation of bioactive molecules in fraction F4-2 through UF-LC-MS/MS combined with metabolomics tools and bioresource searching.

Number	Average Retention Time (min)	Average M/Z	Adduct Ion Name	Area Ratio (4-2P/4-2B)	Predicted Formula	Compound Name	Annotation Method	MS/MS Matched (Y/N)	Bioresource	Reports on Anti-Gout or Anti-Inflammatory Activities
Compound **1**	19.92	233.1533	[M+H]^+^	---(Area of 4-2B is 0)	C_15_H_22_O_3_	Chondroterpene C	MW-MF searching	N	*P. capillacea* symbiotic fungus	effectively inhibits the production of NO in BV-2 cells stimulated by LPS [39]
Compound **2**	11.28	247.1686	[M+H]^+^	---(Area of 4-2B is 0)	C_16_H_24_O_3_	Chondroterpene H	MW-MF searching	N	*P. capillacea* symbiotic fungus [39]	-
Compound **3**	14.97	637.3047	[M+H]^+^	23.67	C_29_H_48_O_15_	[6-[4-acetyloxy-3-hydroxy-2,5-bis(hydroxymethyl)oxolan-2-yl]oxy-2-(hydroxymethyl)-4,5-di(pentanoyloxy)oxan-3-yl] pentanoate	MW-MF searching, MS/MS matching (MSDIAL, FBMN)	Y	*Nicotiana tabacum* [40]	*-*
Compound **4**	17.17	223.2052	[M+H]^+^	23.02	C_15_H_26_O	unknown	MW-MF searching, MS/MS matching (MSDIAL, FBMN)	Y	*Euglena gracilis* [41]	-
Compound **5**	11.98	373.1336	[M+H]^+^	12.85	C_20_H_20_O_7_	Sinensetin	MW-MF searching, MS/MS matching (MSDIAL, FBMN)	Y	*Citrus tankan, Citrus keraji* [42]	anti-inflammatory and anti-oxidative activities [43]
Compound **6**	15.97	277.2161	[M+H]^+^	9.39	C_18_H_28_O_2_	Octadeca-2,4,6,8-tetraenoic acid	MW-MF searching, MS/MS matching (MSDIAL, MSFINDER, FBMN)	Y	*Cystoclonium purpureum* [44]	-
Compound **7**	14.96	243.1008	[M+H]^+^	7.73	C_15_H_14_O_3_	Fenoprofen	MW-MF searching, MS/MS matching (MSDIAL, MSFINDER, FBMN)	N	unknown	non-steroidal drug used for acute pain and chronic arthritis [45]
Compound **8**	17.39	274.2743	[M+H-H_2_O]^+^	1.91	C_16_H_32_O_2_	Palmitic acid	MW-MF searching, MS/MS matching (MSDIAL, FBMN)	Y	*Ulva lactuca* [46] *Pterocladiella tenuis* (NPASS)	palmitic acid in propolis exhibits XO-inhibitory activity [47]
Compound **9**	14.89	263.1275	[M+H]^+^	1.77	C_15_H_18_O_4_	Chondroterpene B	MW-MF searching	N	*P. capillacea* symbiotic fungus [39]	-
Compound **10**	0.72	235.1689	[M+H]^+^	1.31	C_15_H_24_O_3_	Chondroterpene E	MW-MF searching	N	*P. capillacea* symbiotic fungus [39]	-
Compound **11**	14.97	338.0263	[M+H]^+^	1.55	C_13_H_11_N_3_O_4_S_2_	Tenoxicam	MW-MF searching, MS/MS matching (MSDIAL, MSFINDER, FBMN)	Y	unknown	used as drug to treat pain and inflammation in osteoarthritis and rheumatoid arthritis [48]

Note: MW-MF searching: molecular weight (MW) and molecular formula (MF) searching in multiple natural product databases. Y: Yes; N: No. The structures of these compounds are presented in Figure 5.

**Table 2:** The annotation of bioactive molecules in fraction F4-3 through UF-LC-MS/MS combined with metabolomics tools and bioresource searching.

Number	Average Retention Time (min)	Average M/Z	Adduct Ion Name	Area Ratio (4-2P/4-2B)	Predicted Formula	Compound Name	Annotation Method	MS/MS Matched (Y/N)	Bioresource	Reports on Anti-Gout or Anti-Inflammatory Activities
Compound **12**	12.86	300.2905	[M+H]^+^	35.22	C_18_H_37_NO_2_	C_18_-sphingosine	MW-MF searching, MS/MS matching (MSDIAL, MSFINDER, FBMN)	Y	*Amansia glomerata*, *Laurencia nidifica* [49]	anti-inflammatory activity [50]
Compound **13**	0.91	221.1241	[M+H]^+^	21.12	C_13_H_16_O_3_	Precocene ii	MW-MF searching, MS/MS matching (MSDIAL, MSFINDER, FBMN)	Y	*Artemisia capillaris*, *Boenninghausenia albiflora* (LOTUS)	antibacterial and antioxidant activities [51,52]
Compound **14**	11.97	385.0913	[M+Na]^+^	20.07	C_18_H_18_O_8_	Methyl Asterrate	MW-MF searching, MS/MS matching (MSDIAL, MSFINDER, FBMN)	Y	*Ruprechtia tangarana* (COCONUT)	-
Compound **15**	11.97	577.1323	[M+K]^+^	16.89	C_25_H_30_O_13_	Grandifloroside	MW-MF searching, MS/MS matching (MSDIAL, MSFINDER, FBMN)	Y	*Neonauclea sessilifolia* (PubChem)	used in traditional medicine to treat gout [53]
Compound **16**	15.02	670.3364	[2M+ACN+H]^+^	6950.76	unknown	unknown	MW-MF searching, MS/MS matching (MSFINDER)	N	unknown	-
Compound **17**	14.99	295.1728	[M+Na]^+^	13.80	C_18_H_24_O_2_	Estradiol	MW-MF searching, MS/MS matching (MSDIAL, MSFINDER, FBMN)	Y	*Punica granatum* (LOTUS)	reduces serum urate levels [54]
Compound **18**	15.11	241.1587	[M+Na]^+^	11.67	C_15_H_22_O	Germacrone	MW-MF searching, MS/MS matching (MSDIAL, FBMN)	Y	*Curcuma amada*, *Curcuma aeruginosa* (COCONUT)	reduces serum uric acid levels in mice in diabetes-related studies [55]
Compound **19**	13.27	369.1823	[M+Na]^+^	9.99	C_19_H_22_O_6_	Cynaropicrin	MW-MF searching, MS/MS matching (MSDIAL, FBMN)	Y	*Centaurea scoparia* (NPASS)	showes a defensive mechanism against oxidative stress and neuroinflammation by inhibiting the NF-κB pathway. [56]
Compound **20**	17.17	258.2422	[M+NH_4_]^+^	23.02	C_15_H_28_O_2_	Proximadiol	MW-MF searching, MS/MS matching (MSDIAL, MSFINDER, FBMN)	Y	*Euglena gracilis* (LOTUS)	-
Compound **7**	12.18	243.1013	[M+H]^+^	1.88	C_15_H_14_O_3_	Fenoprofen	MW-MF searching, MS/MS matching (MSDIAL, MSFINDER, FBMN)	Y	unknown	non-steroidal drug used for acute pain and chronic arthritis [45]
Compound **8**	12.60	274.2735	[M+H-H_2_O]^+^	1.91	C_16_H_32_O_2_	Palmitic acid	MW-MF searching, MS/MS matching (MSDIAL, MSFINDER, FBMN)	Y	*Ulva lactuca* (LOTUS)	palmitic acid in propolis exhibits XOD-inhibitory activity [47]

Note: MW-MF searching: molecular weight (MW) and molecular formula (MF) searching in multiple natural product databases. Y: Yes; N: No. The structures of these compounds are presented in Figure 6.

**Table 3:** The docking results of 7 annotated compounds with XOD crystal structure (PDB ID: 3nvw).

PubChem ID	Compound Name	Hydrogen-Bonding Residues	Minimum Binding Affinity (kcal/mol)
139589725	Chondroterpene B	THR-1083, SER-1080, SER-1082	−8.4
139589728	Chondroterpene E	SER-1080, THR-1083, SER-1082, GLN-1040	−7.8
165258	Proximadiol	GLN-1194	−7.5
11778225	Octadeca-2,4,6,8-tetraenoic acid	ARG-880, THR-1010	−7.4
139589726	Chondroterpene C	TRY-735, ILE-698	−6.8
139589731	Chondroterpene H	HIS-1212	−6.3
1104	Sphingosine	—	—

**Table 4:** The results of ADMET analysis of the annotated compounds.

Name	Chondroterpene C	Chondroterpene H	Proximadiol	Octadeca-2,4,6,8-Tetraenoic Acid	Chondroterpene B	Chondroterpene E	C_18_-Sphingosine
MW	250.160	264.170	240.210	276.210	254.190	252.170	299.280
LogS	−2.596	−3.083	−1.99	−3.054	−2.835	−1.967	−4.193
HIA	0.019	0.023	0.006	0.019	0.029	0.045	0.314
PPB	62.65%	64.28%	84.36%	95.56%	58.72%	38.63%	98.30%
BBB	0.932	0.859	0.875	0.013	0.982	0.969	0.167
CYP1A2-inhibitor	0.017	0.008	0.022	0.459	0.021	0.007	0.317
CYP2C19-inhibitor	0.038	0.139	0.028	0.26	0.013	0.049	0.249
CYP2C9-inhibitor	0.02	0.108	0.107	0.588	0.03	0.049	0.103
CYP2D6-inhibitor	0.007	0.011	0.009	0.849	0.003	0.006	0.483
CL	9.426	11.781	8.425	0.957	13.004	11.913	3.769
H-HT	0.283	0.221	0.049	0.449	0.332	0.246	0.358
DILI	0.048	0.148	0.026	0.552	0.039	0.091	0.02
Skin Sensitization	0.041	0.297	0.096	0.959	0.061	0.061	0.928
Lipinski Rule	Accepted	Accepted	Accepted	Accepted	Accepted	Accepted	Accepted
Pfizer Rule	Accepted	Accepted	Rejected	Rejected	Accepted	Accepted	Rejected
GSK Rule	Accepted	Accepted	Accepted	Rejected	Accepted	Accepted	Rejected
Golden Triangle	Accepted	Accepted	Accepted	Accepted	Accepted	Accepted	Accepted

## Data Availability

All data are provided in full in the results section and Supplementary Materials of this paper.

## Supplementary Materials

The following supporting information can be downloaded at: https://www.mdpi.com/article/10.3390/md21100502/s1, Figure S1: The MS/MS spectrum of compound 1 captured by the affinity ultrafiltration from fraction F4-2(P); Figure S2: The MS/MS spectrum of compound 2 captured by the affinity ultrafiltration from fraction F4-2(P); Figure S3: The MS/MS spectrum of the [M+NH4]+ ion of compound 3 captured by the affinity ultrafiltration from fraction F4-2(P); Figure S4: The MS/MS spectrum of the [M+H]+ ion of compound 3 captured by the affinity ultrafiltration from fraction F4-2(P); Figure S5: The MS/MS spectrum of compound 4 captured by the affinity ultrafiltration from fraction F4-2(P); Figure S6: The MS/MS spectrum of compound 5 captured by the affinity ultrafiltration from fraction F4-2(P); Figure S7: The MS/MS spectrum of compound 6 captured by the affinity ultrafiltration from fraction F4-2(P); Figure S8: The MS/MS spectrum of compound 7 captured by the affinity ultrafiltration from fractions F4-2(P) and F4-3(P); Figure S9: The MS/MS spectrum of compound 8 captured by the affinity ultrafiltration from fractions F4-2(P) and F4-3(P); Figure S10: The MS/MS spectrum of compound 9 captured by the affinity ultrafiltration from fraction F4-2(P); Figure S11: The MS/MS spectrum of compound 10 captured by the affinity ultrafiltration from fraction F4-2(P); Figure S12: The MS/MS spectrum of compound 11 captured by the affinity ultrafiltration from fraction F4-2(P); Figure S13: The MS/MS spectrum of compound 12 captured by the affinity ultrafiltration from fraction F4-3(P); Figure S14: The MS/MS spectrum of compound 13 captured by the affinity ultrafiltration from fraction F4-3(P); Figure S15: The MS/MS spectrum of compound 14 captured by the affinity ultrafiltration from fraction F4-3(P); Figure S16: The MS/MS spectrum of compound 15 captured by the affinity ultrafiltration from fraction F4-3(P); Figure S17: The MS/MS spectrum of compound 16 captured by the affinity ultrafiltration from fraction F4-3(P); Figure S18: The MS/MS spectrum of compound 17 captured by the affinity ultrafiltration from fraction F4-3(P); Figure S19: The MS/MS spectrum of compound 18 captured by the affinity ultrafiltration from fraction F4-3(P); Figure S20: The MS/MS spectrum of compound 19 captured by the affinity ultrafiltration from fraction F4-3(P); Figure S21: The MS/MS spectrum of compound 20 captured by the affinity ultrafiltration from fraction F4-3(P).

## References

[1] Kwon H.C., Ahn S.S., Yoo B.W., Yoo J., Jung S.M., Song J.J., Park Y.B., Lee S.W. (2020). Hyperuricemia is associated with decreased renal function and occurrence of end-stage renal disease in patients with microscopic polyangiitis and granulomatosis with polyangiitis: A retrospective study. Rheumatol. Int..

[2] So A.K., Martinon F. (2017). Inflammation in gout: Mechanisms and therapeutic targets. Nat. Rev. Rheumatol..

[3] Choi H.K., Mount D.B., Reginato A.M. (2005). Pathogenesis of Gout. Ann. Intern. Med..

[4] Wang Y.T., Zhang W.L., Qian T.T., Sun H., Xu Q. (2021). Reduced renal function may explain the higher prevalence of hyperuricemia in older people. Sci. Rep..

[5] Ruiz-Miyazawa K.W., Borghi S.M., Pinho-Ribeiro F.A., Staurengo-Ferrari L., Fattori V., Fernandes G.S.A., Casella A.M., Alves-Filho J.C., Cunha T.M., Cunha F.Q. (2017). Quercetin inhibits gout arthritis in mice: Induction of an opioid-dependent regulation of inflammasome. Inflammopharmacology.

[6] Barros C.H., Matosinhos R.C. (2021). *Lychnophora pinaster*’s effects on inflammation and pain in acute gout. J. Ethnopharmacol..

[7] Hille R., Hall J., Basu P. (1996). The mononuclear molybdenum enzymes. Chem. Rev..

[8] Zhao M.M. (2014). In Vitro and In Vivo studies on adlay-derived seed extracts: Phenolic profiles, antioxidant activities, serum uric acid suppression, and xanthine oxidase inhibitory effects. J. Agric. Food Chem..

[9] Lee B.E., Toledo A.H., Anaya-Prado R., Roach R.R., Toledo-Pereyra L.H. (2009). Allopurinol, xanthine oxidase, and cardiac ischemia. J. Investig. Med. Off. Publ. Am. Fed. Clin. Res..

[10] Bruce S.P. (2006). Febuxostat: A selective xanthine oxidase inhibitor for the treatment of hyperuricemia and gout. Ann. Pharmacother..

[11] Takashi N., Takayo M., Mai N., Nobutaka M., Naoki A., Takashi I., Ryusuke S. (2016). Effects of topiroxostat and febuxostat on urinary albumin excretion and plasma xanthine oxidoreductase activity in dbidb mice. Eur. J. Pharmacol. Int. J..

[12] Gliozzi M., Malara N., Muscoli S., Mollace V. (2016). The treatment of hyperuricemia. Int. J. Cardiol..

[13] Badve S.V., Pascoe E.M., Tiku A., Boudville N., Brown F.G., Cass A., Clarke P., Dalbeth N., Day R.O., Zoysa J.R.d. (2020). Effects of Allopurinol on the Progression of Chronic Kidney Disease. N. Engl. J. Med..

[14] Yao F.F., Zhang R., Fu R.J., Chen J., He W. (2012). Effect and mechanism study of the same doses of Quercetin and Apigenin on hyperuricemic rats. Mod. Prev. Med..

[15] Zhu J.X., Wang Y., Kong L.D., Yang C., Zhang X. (2004). Effects of *Biota orientalis* extract and its flavonoid constituents, quercetin and rutin on serum uric acid levels in oxonate-induced mice and xanthine dehydrogenase and xanthine oxidase activities in mouse liver. J. Ethnopharmacol..

[16] Shin-Ichi A., Mifuyu O., Shinji K., Kazumi Y. (2021). Comparative effects of quercetin, luteolin, apigenin and their related polyphenols on uric acid production in cultured hepatocytes and suppression of purine bodies-induced hyperuricemia by rutin in mice. Cytotechnology.

[17] Ou R.R., Lin L.Z., Zhao M.M., Xie Z.Q. (2020). Action mechanisms and interaction of two key xanthine oxidase inhibitors in galangal: Combination of In Vitro and in silico molecular docking studies. Int. J. Biol. Macromol..

[18] Zhang C., Zhang G.W., Pan J.H., Gong D.M. (2016). Galangin competitively inhibits xanthine oxidase by a ping-pong mechanism. Food Res. Int..

[19] Liang Q.C., Shen G.Z., Wei T., Wu D.M. (2011). Study on interaction between myricetin and XO via spectroscopy. J. Harbin Univ. Commer. Nat. Sci. Ed..

[20] Zhang C., Zhang G.W., Liao Y.J. (2017). Myricetin inhibits the generation of superoxide anion by reduced form of xanthine oxidase. Food Chem..

[21] Cengiz S., Yurdakoc K., Aksu S. (2012). Inhibition of xanthine oxidase by Caulerpenyne from *Caulerpa prolifera*. Turk. J. Biochem..

[22] Zhang D.Y., Liu H.Z., Luo P., Li Y.Q. (2018). Production Inhibition and Excretion Promotion of Urate by Fucoidan from *Laminaria japonica* in Adenine-Induced Hyperuricemic Mice. Mar. Drugs.

[23] Zhang Y., Tan X.H., Lin Z., Liu H.Z., Shang J.H. (2021). Fucoidan from *Laminaria japonica* inhibits expression of GLUT9 and URAT1 via PI3K/Akt, JNK and NF-κB pathways in uric acid-exposed HK-2 Cells. Mar. Drugs.

[24] Li X.Q., Gao X.X. (2021). The anti-hyperuricemic effects of green alga *Enteromorpha prolifera* polysaccharide via regulation of the uric acid transporters In Vivo. Food Chem. Toxicol..

[25] Chen G.L., Guo M.Q. (2017). Rapid screening for α-Glucosidase inhibitors from *Gymnema sylvestre* by Affinity Ultrafiltration–HPLC-MS. Front. Pharmacol..

[26] Tsugawa H., Cajka T., Kind T., Ma Y. (2015). MS-DIAL: Data-independent MS/MS deconvolution for comprehensive metabolome analysis. Nat. Methods.

[27] Tsugawa H. (2020). Computational MS/MS fragmentation and structure elucidation using MS-FINDER software. Compr. Nat. Prod. III.

[28] Oppong-Danquah E., Parrot D., Bluemel M., Labes A., Tasdemir D. (2018). Molecular networking-based metabolome and bioactivity analyses of marine-adapted fungi co-cultivated with phytopathogens. Front. Microbiol..

[29] Nothias L.F., Petras D., Schmid R., Dührkop K., Rainer J. (2020). Feature-based Molecular Networking in the GNPS analysis environment. Nat. Methods.

[30] Remy S., Solis D., Silland P., Neyts J., Roussi F., Touboul D., Litaudon M. (2019). Isolation of phenanthrenes and identification of phorbol ester derivatives as potential anti-CHIKV agents using FBMN and NAP from *Sagotia racemosa*. Phytochemistry.

[31] Nie Y.Y., Yang W.C., Liu Y.Y., Yang J.M., Lei X.L., Gerwick W.H., Zhang Y. (2020). Acetylcholinesterase inhibitors and antioxidants mining from marine fungi: Bioassays, bioactivity coupled LC–MS/MS analyses and molecular networking. Mar. Life Sci. Technol..

[32] Patarra R.F., Iha C., Pereira L., Neto A.I. (2020). Concise review of the species *Pterocladiella capillacea* (S.G. Gmelin) Santelices & Hommersand. J. Appl. Phycol..

[33] Cavalli P.A., Wanderlind E.H., Hemmer J.V., Gerlach O.M.S., Emmerich A.K., Bella-Cruz A., Tamanahae M.r., Almerindo G.I. (2021). *Pterocladiella capillacea*-stabilized silver nanoparticles as a green approach toward antibacterial biomaterials. New J. Chem..

[34] Alencar D.B.D., Diniz J.C., Rocha S.A.S., Pires-Cavalcante K.M.S., Lima R.L.D., Sousa K.C.D., Freitas J.O., Bezerra R.M., Baracho B.M., Sampaio A.H. (2018). Fatty acid composition from the marine red algae *Pterocladiella capillacea* (S. G. Gmelin) Santelices & Hommersand 1997 and *Osmundaria obtusiloba* (C. Agardh) R. E. Norris 1991 and its antioxidant activity. An. Acad. Bras. Ciências.

[35] Alencar D.B.d., Carvalho F.C.T.d., Rebouças R.H., Santos D.R.D., Pires-Cavalcante K.M.D.S., Lima R.L.d., Baracho B.M., Bezerra R.M., Viana F.A., Vieira R.H.S.D.F. (2016). Bioactive extracts of red seaweeds *Pterocladiella capillacea* and *Osmundaria obtusiloba* (Floridophyceae:Rhodophyta) with antioxidant and bacterial agglutination potential. Asian Pac. J. Trop. Med..

[36] Bou-Salah L., Benarous K., Linani A., Rabhi F., Chaib K., Chine I., Bensaidane H., Yousfi M. (2021). Anti-inflammatory drugs as new inhibitors to xanthine oxidase: In vitro and in silico approach. Mol. Cell. Probes.

[37] Nile S.H., Ko E.Y., Kim D.H., Keum Y.-S. (2015). Screening of ferulic acid related compounds as inhibitors of xanthine oxidase and cyclooxygenase-2 with anti-inflammatory activity. Rev. Bras. Farmacogn..

[38] Preethi J., Chitra L., Ancy I., Kumaradhas P., Palvannan T. (2018). S-allyl cysteine as potent anti-gout drug: Insight into the xanthine oxidase inhibition and anti-inflammatory activity. Biochimie.

[39] Hsiao G., Chi W.C., Pang K.L., Chen J., Kuo Y. (2017). Hirsutane-Type sesquiterpenes with inhibitory activity of microglial nitric oxide production from the red alga-derived fungus *Chondrostereum* sp. NTOU4196. J. Nat. Prod..

[40] Tsugawa H., Nakabayashi R., Mori T., Yamada Y., Takahashi M. (2019). A cheminformatics approach to characterize metabolomes in stable-isotope-labeled organisms. Nat. Methods.

[41] He J.Y., Liu C.C., Du M.Z., Zhou X.Y., Hu Z.L., Lei A.P., Wang J.X. (2021). Metabolic responses of a model green microalga *Euglena gracilis* to different environmental stresses. Front. Bioeng. Biotechnol..

[42] Chen J., Montanari A.M., Widmer W.W. (1997). Two new polymethoxylated flavones, a class of compounds with potential anticancer activity, isolated from cold pressed dancy tangerine peel oil solids. J. Agric. Food Chem..

[43] Yang D., Yang R.G., Shen J.Y., Huang L., Men S., Wang T.C. (2021). Sinensetin attenuates oxygen-glucose deprivation/reperfusion-induced neurotoxicity by MAPK pathway in human cerebral microvascular endothelial cells. J. Appl. Toxicol. JAT.

[44] Findwy J.A., Patil A.D. (1986). Antibacterial constituents of the red alga *Cystoclonium purpureum*. Phytochemistry.

[45] Useini L., Mojić M., Laube M., Lönnecke P., Mijatović S. (2023). Carborane analogues of Fenoprofen exhibit Improved antitumor activity. ChemMedChem.

[46] Ashry E.S.H.E., Atta-ur-Rahman, Choudhary M.I., Kandil S.H., Nemr A.E., Gulzar T., Shobier A.H. (2011). Studies on the constituents of the green alga *Ulva lactuca*. Chem. Nat. Compd..

[47] Naik R.R., Shakya A.K., Oriquat G.A., Katekhaye S. (2021). Fatty acid analysis, chemical constituents, biological activity and pesticide residues screening in jordanian propolis. Molecules.

[48] Elakkad Y.E., Younis M.K., Allam R.M., Mohsen A.F., Khalil I.A. (2021). Tenoxicam loaded hyalcubosomes for osteoarthritis. Int. J. Pharm..

[49] Ardellina J.H., Moore R.E. (1978). Sphingosine derivatives from red algae of the ceramiales. Phytochemistry.

[50] Verstockt B., Vetrano S., Salas A., Nayeri S., Duijvestein M., Casteele N.V. (2022). Sphingosine 1-phosphate modulation and immune cell trafficking in inflammatory bowel disease. Nat. Rev. Gastroenterol. Hepatol..

[51] Furukawa T., Sakamoto N., Suzuki M., Kimura M., Nagasawa H., Sakuda S. (2015). Precocene II, a trichothecene production inhibitor, binds to voltage-dependent anion channel and increases the superoxide level in mitochondria of *Fusarium graminearum*. PLoS ONE.

[52] Sukmawan Y.P., Anggadiredja K., Adnyana I.K. (2023). Anti-neuropathic pain mechanistic study on *A. conyzoides* essential oil, Precocene II, Caryophyllene, or Longifolene as single agents and in combination with pregabalin. CNS Neurol. Disord.—Drug Targets.

[53] Anyanwu G.O., Nisar-ur-Rehman, Onyeneke C.E., Rauf K. (2015). Medicinal plants of the genus Anthocleista—A review of their ethnobotany, phytochemistry and pharmacology. J. Ethnopharmacol..

[54] Liu L., Zhao T.Y., Shan L.Z., Cao L., Zhu X.X., Xue Y. (2021). Estradiol regulates intestinal ABCG2 to promote urate excretion via the PI3K/Akt pathway. Nutr. Metab..

[55] Wang Y.G., Feng F.F., He W.F., Sun L.F., He Q., Jin J. (2022). MiR-188-3p abolishes germacrone-mediated podocyte protection in a mouse model of diabetic nephropathy in type I diabetes through triggering mitochondrial injury. Bioengineered.

[56] Jin T., Leng B. (2023). Cynaropicrin averts the oxidative stress and neuroinfammation in ischemic/reperfusion injury through the modulation of NF-κB. Appl. Biochem. Biotechnol..

[57] Yamaguchi Y., Matsumura T., Ichida K., Okamoto K., Nishino T. (2007). Human xanthine oxidase changes its substrate specificity to aldehyde oxidase type upon mutation of amino acid residues in the active site: Roles of active site residues in binding and activation of purine substrate. J. Biochem..

[58] Lipinski C.A., Lombardo F., Dominy B.W., Feeney P.J. (2001). Experimental and computational approaches to estimate solubility and permeability in drug discovery and development settings. Adv. Drug Deliv. Rev..

[59] Ma K., Bao L., Han J.J., Jin T., Yang X.L., Zhao F., Li S.F., Song F.H., Liu M.M., Liu H.W. (2014). New benzoate derivatives and hirsutane type sesquiterpenoids with antimicrobial activity and cytotoxicity from the solid-state fermented rice by the medicinal mushroom *Stereum hirsutum*. Food Chem..

[60] Qi Q.Y., Bao L., Ren J.W., Han J.J., Zhang Z.Y., Li Y., Yao Y.J., Cao R., Liu H.W. (2014). Sterhirsutins A and B, two new heterodimeric sesquiterpenes with a new skeleton from the culture of *Stereum hirsutum* collected in Tibet Plateau. Org. Lett..

[61] Huang L., Lan W.J., Li H.J. (2018). Two new hirsutane-type sesquiterpenoids chondrosterins N and O from the marine fungus *Chondrostereum* sp.. Nat. Prod. Res..

[62] Huang L., Lan W.J., Deng R., Feng G.K., Xu Q.Y., Hu Z.Y. (2016). Additional new cytotoxic triquinane-type sesquiterpenoids chondrosterins K-M from the marine fungus *Chondrostereum* sp.. Mar. Drugs.

[63] Liu H.X., Tan H.B., Chen K., Chen Y.C., Li S.N., Li H.H., Zhang W.M. (2018). Cerrenins A-C, cerapicane and isohirsutane sesquiterpenoids from the endophytic fungus *Cerrena* sp.. Fitoterapia.

[64] Cha H.J., Chiang M.W.L., Guo S.Y., Lin S.M., Pang K.L. (2021). Culturable fungal community of *Pterocladiella capillacea* in Keelung, Taiwan: Effects of surface sterilization method and isolation medium. J. Fungi.

[65] Tiwari P., Bae H. (2022). Endophytic Fungi: Key insights, emerging prospects, and challenges in natural product drug discovery. Microorganisms.

[66] Tang P., Si S., Liu L. (2015). Analysis of bovine serum albumin ligands from *Puerariae flos* using ultrafiltration combined with HPLC-MS. J. Chem..

[67] Wang J., Liu S., Ma B., Chen L. (2014). Rapid screening and detection of XOD inhibitors from *S. tamariscina* by ultrafiltration LC-PDA-ESI-MS combined with HPCCC. Anal. Bioanal. Chem..

[68] Chen G.L., Huang B.X., Guo M.Q. (2018). Current advances in screening for bioactive components from medicinal plants by affinity ultrafiltration mass spectrometry. Phytochem. Anal. PCA.

[69] Wang M.X., Carver J.J., Phelan V.V., Sanchez L.M. (2016). Sharing and community curation of mass spectrometry data with Global Natural Products Social Molecular Networking. Nat. Biotechnol..

[70] Andriana Y., Xuan T.D., Quy T.N., Minh T.N., Van T.M., Viet T.D. (2019). Antihyperuricemia, Antioxidant, and Antibacterial Activities of *Tridax procumbens* L.. Foods.

[71] Chen M.Q., Liang J.Y., Wang Y., Liu Y., Zhou C.X., Hong P.Z., Zhang Y., Qian Z.J. (2022). A new benzaldehyde from the coral-derived fungus *Aspergillus* terreus C23-3 and its anti-Inflammatory effects via suppression of MAPK signaling pathway in RAW264.7 cells. J. Zhejiang Univ.-Sci. B Biomed. Biotechnol..

[72] Sun Y., Liu W.C., Shi X., Zheng H.Z., Zheng Z., Lu X.H., Xing Y. (2021). Inducing secondary metabolite production of *Aspergillus sydowii* through microbial co-culture with *Bacillus subtilis*. Microb. Cell Factories.

[73] Triastuti A., Haddad M., Barakat F., Mejia K., Rabouille G., Fabre N., Amasifuen C., Jargeat P., Vansteelandt M. (2021). Dynamics of chemical diversity during co-cultures: An integrative time-scale metabolomics study of fungal Endophytes *Cophinforma mamane* and *Fusarium solani*. Chem. Biodivers..

