# Supplementary material for: Mining Xanthine Oxidase Inhibitors from an Edible Seaweed Pterocladiella capillacea by Using In Vitro Bioassays, Affinity Ultrafiltration LC-MS/MS, Metabolomics Tools, and In Silico Prediction
Source: Mar Drugs. 2023 Sep 22;21(10):502. doi: 10.3390/md21100502 (PMC10608504; doi:10.3390/md21100502)
Supplement: Supplementary file 1 [file marinedrugs-21-00502-s001.zip › marinedrugs-2594247-supplementary.pdf]

Supplementary Materials

Mining Xanthine Oxidase Inhibitors from an Edible Seaweed *Pterocladiella capillacea* by Using in vitro Bioassays, Affinity Ultrafiltration LC-MS/MS, Metabolomics Tools, and In Silico Prediction

Yawen Wang[1#], Longjian Zhou[1,2,3#], Minqi Chen[1], Yayue Liu[1,2,3], Yu Yang [1], Tiantian Lu[1], Fangfang Ban[1], Xueqiong Hu[1], Zhongji Qian[1,2], Pengzhi Hong[1,2,3], Yi Zhang[1,2,3]*

1. Guangdong Provincial Key Laboratory of Aquatic Product Processing and Safety, Guangdong Provincial Engineering Laboratory for Marine Biological Products, Guangdong Provincial Engineering Technology Research Center of Seafood, Key Laboratory of Advanced Processing of Aquatic Product of Guangdong Higher Education Institution, Guangdong Provincial Center for Modern Agricultural Scientific Innovation, Marine Biomedicine R&D Center at Shenzhen Institute of Guangdong Ocean University, Zhanjiang Municipal Key Laboratory of Marine Drugs and Nutrition for Brain Health, Research Institute for Marine Drugs and Nutrition, College of Food Science and Technology, Guangdong Ocean University, Zhanjiang 524088, China; yavin_wang@163.com (Y.W.); zhoulongjian@gdou.edu.cn (L.Z.); katelyn@outlook.com (M.C.); yayue_liu@163.com (Y.L.); yangyu515900@163.com (Y.Y.); lutiantiana@163.com (T.L.); banfang-fang@126.com (F.B.); hxw247@163.com (X.H.); zjqian78@163.com (Z.Q.); hongpengzhi@126.com (P.H.)

2. Southern Marine Science and Engineering Guangdong Laboratory (Zhanjiang), Zhanjiang 524088, China;

3. Collaborative Innovation Center of Seafood Deep Processing, Dalian Polytechnic University, Dalian 116034, China

[#]These authors have contributed equally to this work.

[*]Corresponding author: Yi Zhang; E-mails: hubeizhangyi@163.com, zhangyi@gdou.edu.cn; Tel:86-759-239-6046. ORCID: 0000-0002-1600-7456.

Figure legends

Figure S1. The MS/MS spectrum of compound **1** captured by the affinity ultrafiltration from fraction F4-2(P).

Figure S2. The MS/MS spectrum of compound **2** captured by the affinity ultrafiltration from fraction F4-2(P).

Figure S3. The MS/MS spectrum of the $[M+NH_4]^+$ ion of compound **3** captured by the affinity ultrafiltration from fraction F4-2(P).

Figure S4. The MS/MS spectrum of the $[M+H]^+$ ion of compound **3** captured by the affinity ultrafiltration from fraction F4-2(P).

Figure S5. The MS/MS spectrum of compound **4** captured by the affinity ultrafiltration from fraction F4-2(P).

Figure S6. The MS/MS spectrum of compound **5** captured by the affinity ultrafiltration from fraction F4-2(P).

Figure S7. The MS/MS spectrum of compound **6** captured by the affinity ultrafiltration from fraction F4-2(P).

Figure S8. The MS/MS spectrum of compound **7** captured by the affinity ultrafiltration from fractions F4-2(P) and F4-3(P).

Figure S9. The MS/MS spectrum of compound **8** captured by the affinity ultrafiltration from fractions F4-2(P) and F4-3(P).

Figure S10. The MS/MS spectrum of compound **9** captured by the affinity ultrafiltration from fraction F4-2(P).

Figure S11. The MS/MS spectrum of compound **10** captured by the affinity ultrafiltration from fraction F4-2(P).

Figure S12. The MS/MS spectrum of compound **11** captured by the affinity ultrafiltration from fraction F4-2(P).

Figure S13. The MS/MS spectrum of compound **12** captured by the affinity ultrafiltration from fraction F4-3(P).

Figure S14. The MS/MS spectrum of compound **13** captured by the affinity ultrafiltration from fraction F4-3(P).

Figure S15. The MS/MS spectrum of compound **14** captured by the affinity ultrafiltration from fraction F4-3(P).

Figure S16. The MS/MS spectrum of compound **15** captured by the affinity ultrafiltration from fraction F4-3(P).

Figure S17. The MS/MS spectrum of compound **16** captured by the affinity ultrafiltration from fraction F4-3(P).

Figure S18. The MS/MS spectrum of compound **17** captured by the affinity ultrafiltration from fraction F4-3(P).

Figure S19. The MS/MS spectrum of compound **18** captured by the affinity ultrafiltration from fraction F4-3(P).

Figure S20. The MS/MS spectrum of compound **19** captured by the affinity ultrafiltration from fraction F4-3(P).

Figure S21. The MS/MS spectrum of compound **20** captured by the affinity ultrafiltration from fraction F4-3(P).

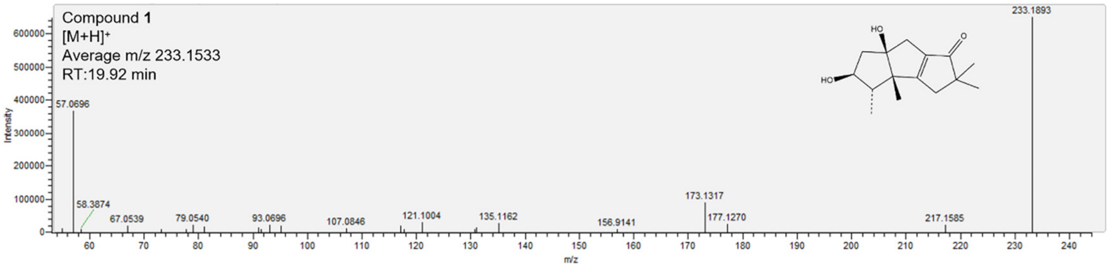

Figure S1

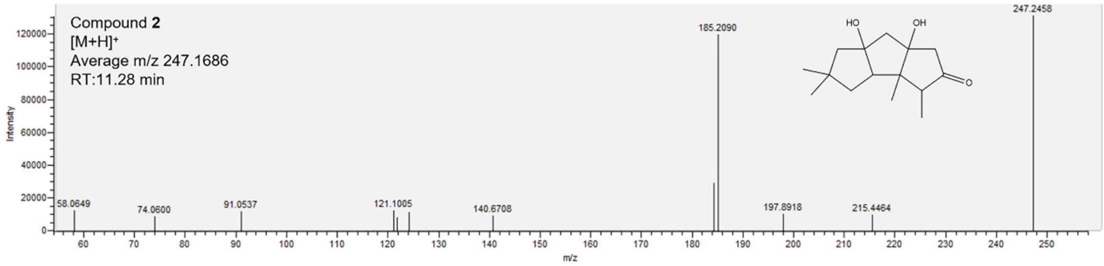

Figure S2

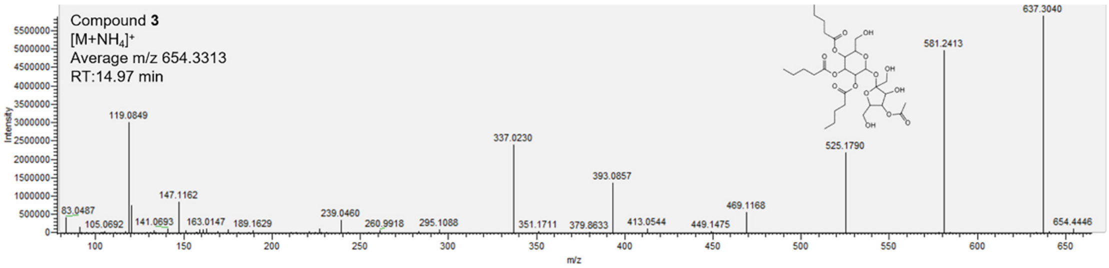

Figure S3

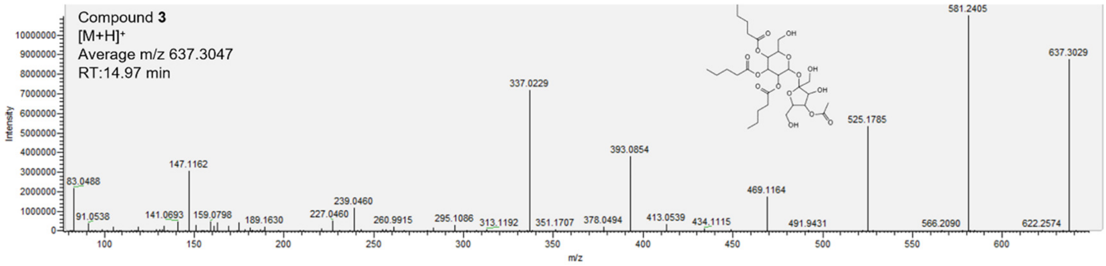

Figure S4

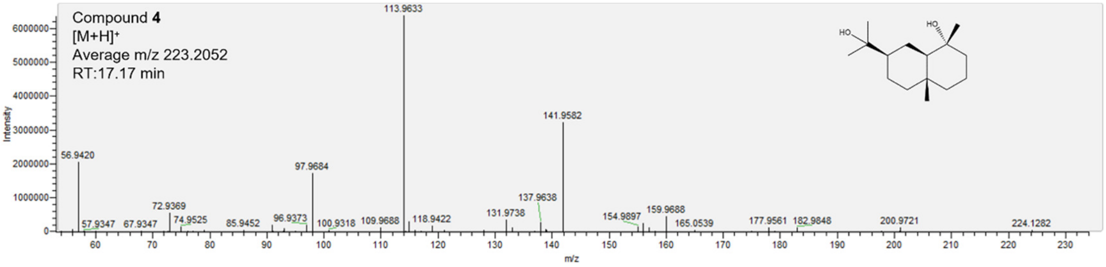

Figure S5

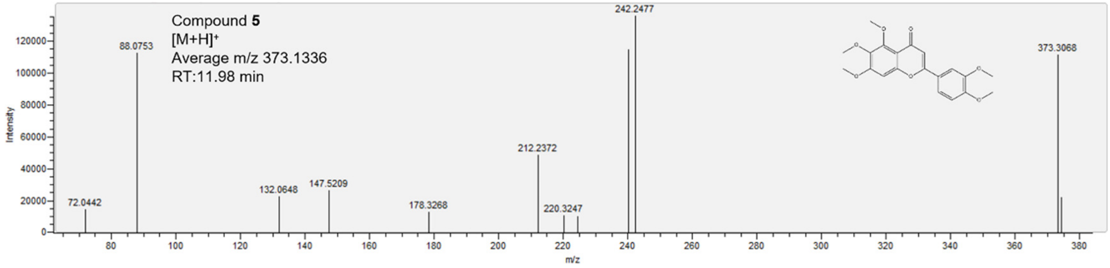

Figure S6

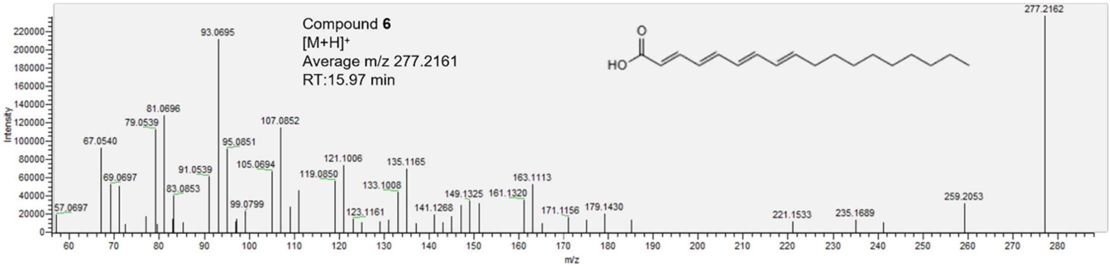

Figure S7

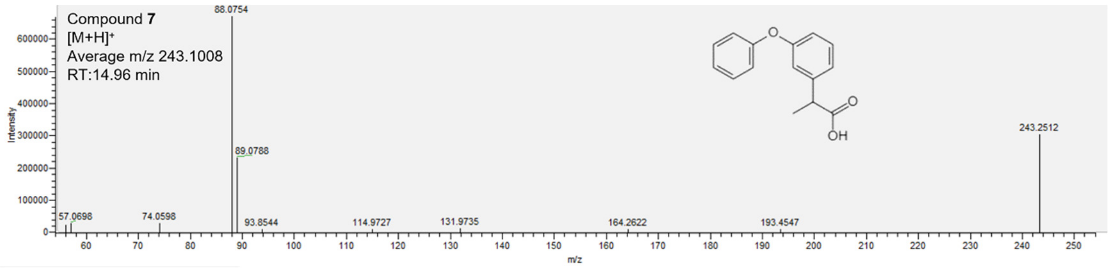

Figure S8

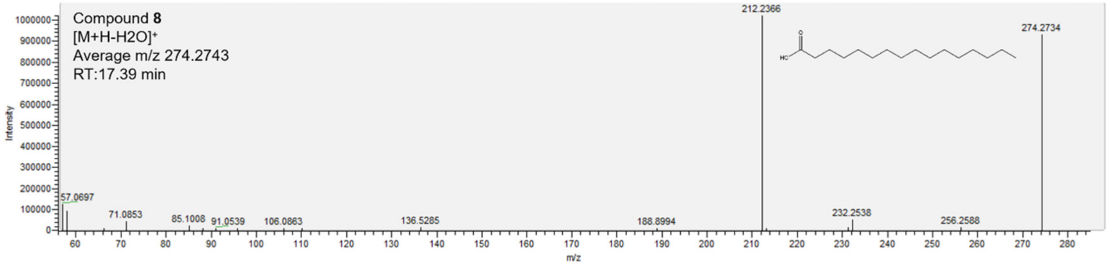

Figure S9

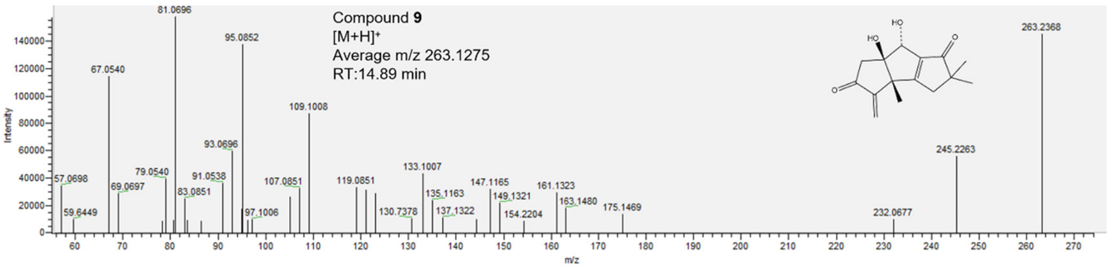

Figure S10

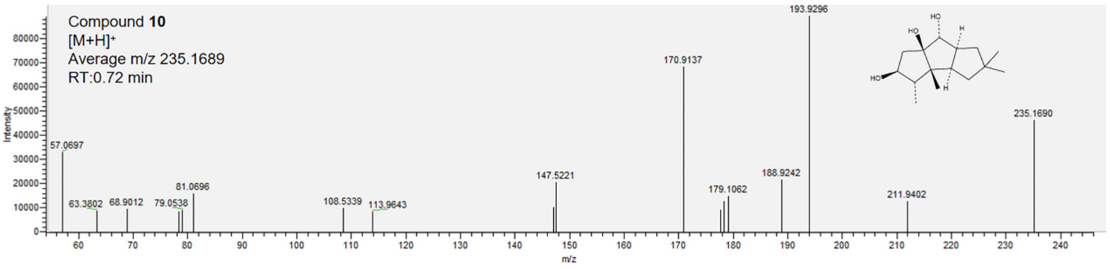

Figure S11

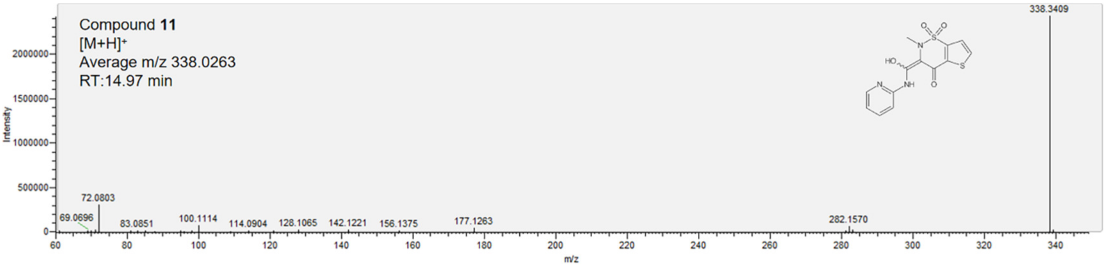

Figure S12

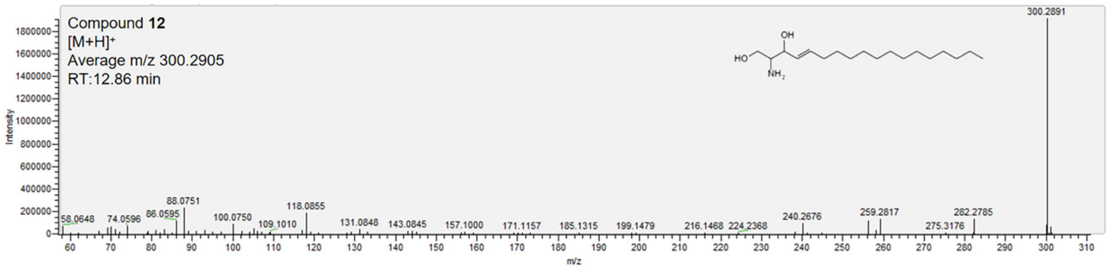

Figure S13

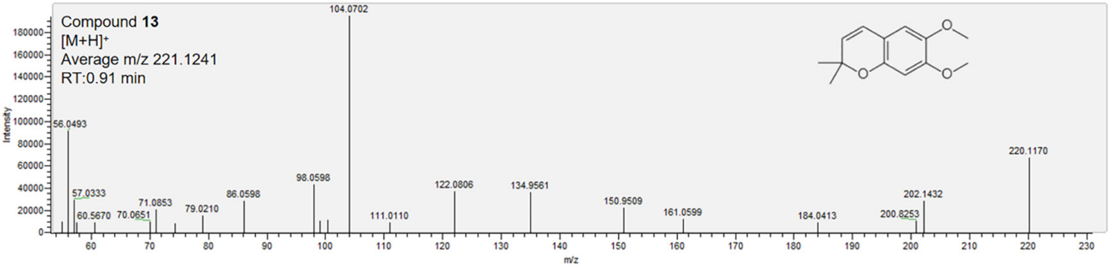

Figure S14

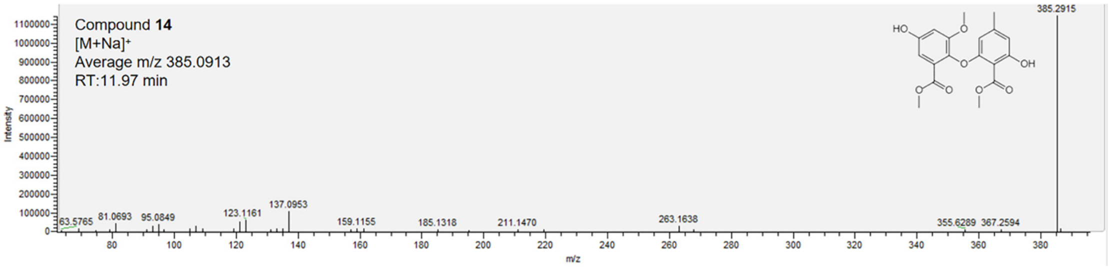

Figure S15

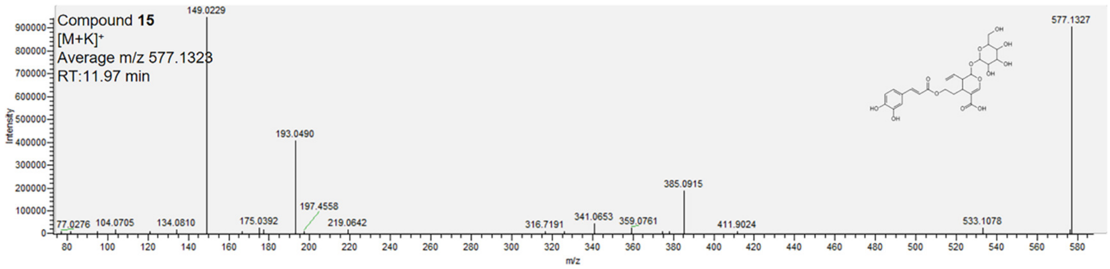

Figure S16

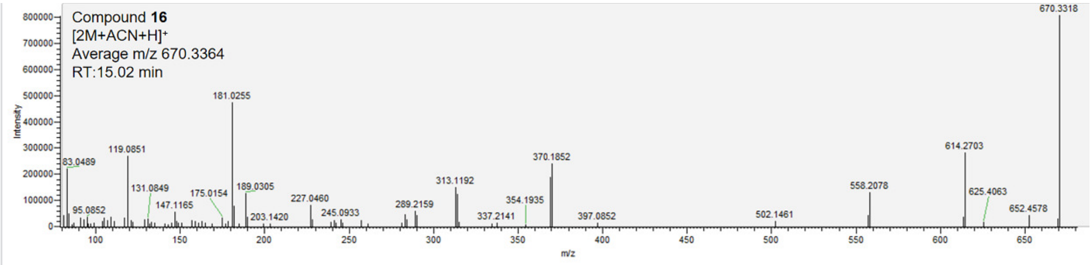

Figure S17

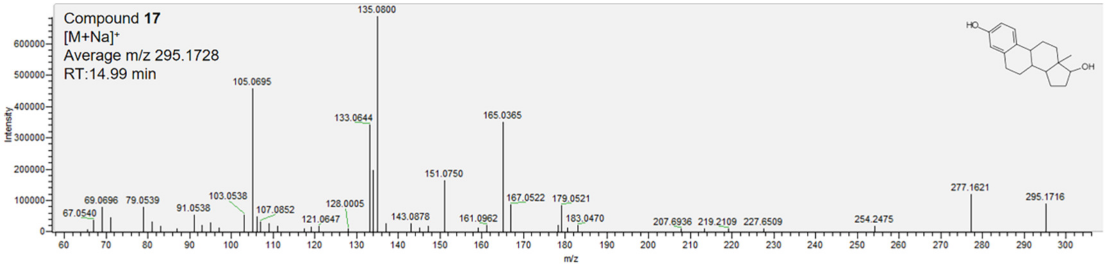

Figure S18

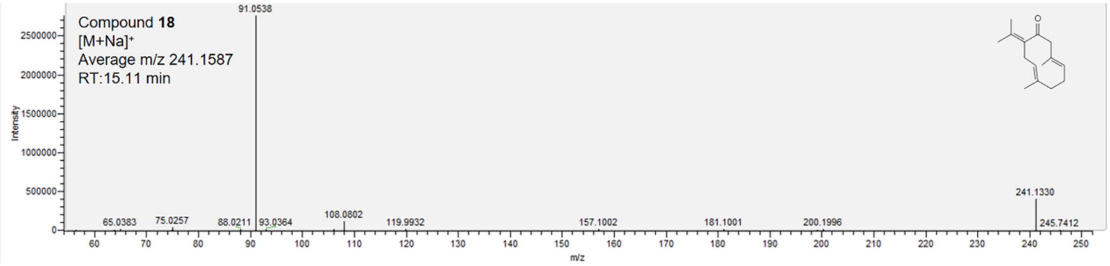

Figure S19

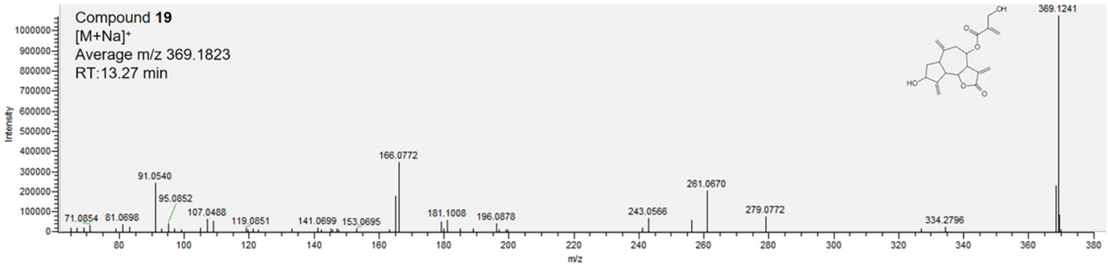

Figure S20

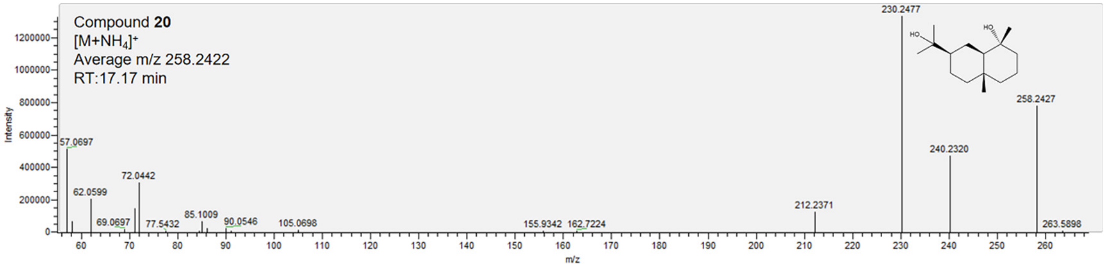

Figure S21
